# Anti-neuropathic effects of *Rosmarinus officinalis* L. terpenoid fraction: relevance of nicotinic receptors

**DOI:** 10.1038/srep34832

**Published:** 2016-10-07

**Authors:** Lorenzo Di Cesare Mannelli, Laura Micheli, Mario Maresca, Giancarlo Cravotto, Maria Bellumori, Marzia Innocenti, Nadia Mulinacci, Carla Ghelardini

**Affiliations:** 1 Dept. of Neuroscience, Psychology, Drug Research and Child Health - NEUROFARBA - Pharmacology and Toxicology Section, University of Florence, Florence, Italy; 2 Dept. Scienza e Tecnologia del Farmaco, University of Turin, Turin, Italy; 3 Dept. of Neuroscience, Psychology, Drug Research and Child Health - NEUROFARBA - Pharmaceutical and Nutraceutical Division, University of Florence, Florence, Italy

## Abstract

Traditional uses and current results highlight the neuroprotective properties of *Rosmarinus officinalis* L. The compelling need for novel strategies able to relieve neuropathic pain encouraged us to analyze different rosemary leaf extracts in rats following chronic constriction injury (CCI) of sciatic nerve. Ethanol, acetone, and the innovative ultrasound-hexane extractive methods were used to obtain: EE, AE, and for hexane extracts URE*prel* and URE. Extracts were characterized in terms of typical constituents and repeatedly administered to CCI-rats (13-days treatment, from the day of surgery). URE showed the best efficacy and potency in reducing hypersensitivity to noxious- and non-noxious stimuli and spontaneous pain. URE contained the higher quantity of the terpenoid carnosic acid (CA) and its efficacy was compared to pure CA. Histological analysis of the sciatic nerve revealed that URE prevented axon and myelin derangement, edema and inflammatory infiltrate. In the dorsal horn of the spinal cord, URE did not reduce astrocyte activation. Both the pain reliever and the neuroconservative effects of URE were significantly prevented by the nicotinic receptor (nAChR) antagonist mecamylamine. In conclusion, the hexane-ultrasound rosemary extract is able to reduce neuropathic hypersensitivity and protect nervous tissues. Effectiveness is mainly related to the terpenoid fraction by mechanisms involving nAChRs.

*Rosmarinus officinalis* L. (Lamiaceae), commonly called rosemary, is woody perennial herb native of the Mediterranean region and now widely spread in European countries. It is a common spice used worldwide for culinary and medicinal purpose^1^, not only for the aromatic fraction but also for the typical phenolic compounds. The hydroalcoholic extracts from *R. officinalis* have been recognized as safe by the European Food Safety Authority (EFSA)^2^ and their use is authorized as natural antioxidants and preservatives of foods. Several biological effects have been associated to rosemary leaf extracts (anti-spasmodic^3^, anti-inflammatory and anti-nociceptive^4^, hepatoprotective^5^, diuretic^6^) due to its various phytochemical constituents. The most represented compounds are monoterpenes (essential oils), diterpene phenols (carnosic acid, carnosol, rosmanol, epirosmanol, isorosmanol), phenolic acids (rosmarinic acid), flavonols and triterpene acids (ursolic acid, oleanolic acid, betulinic acid)^7^,^8^,^9^,^10^,^11^. Among them, carnosic acid (CA) is considered a potent antioxidant useful as protector against free radicals damage together with rosmarinic acid and carnosol^12^,^13^,^14^,^15^. A recent work demonstrated that a hydroalcoholic rosemary extract inhibits acetylcholinesterase activity, improving memory impairment in the rat^16^ furthermore, rosemary polyphenols enhanced cholinergic activities in PC12 cells^17^,^18^. The cholinergic properties of rosemary extracts prompt us to evaluate a possible role in neuropathy treatment since the relevance of the cholinergic system modulation in pain signalling^19^. The efficacy against acute pain is most closely related to the muscarinic component^20^,^21^, on the contrary the cholinergic relevance in neuropathic pain management is imputable to the nicotinic acetylcholine receptors (nAChRs)^22^. Both the anti-hypersensitive and neuroprotective effects of the ACh synthesis promoter acetyl-l-carnitine are blocked by mecamylamine, a nonselective nAChR antagonist^22^. Moreover, selective modulators of the α7^23^ and the α9α10^24^ nAChR subtypes relieved nerve trauma-induced pain in the rat and prevented nervous system derangement that underlie neuropathies.

Aim of this study was to investigate the efficacy of novel extracts of *Rosmarinus officinalis* leaves on Chronic Constriction Injury (CCI) induced neuropathic pain in the rat, a model of trauma-induced neuropathy representative of compressive damage. Different products were obtained by ethanol- (EE), acetone- (AE) or *n*-hexane-ultrasound assisted-extraction (URE*prel* and URE) to compare properties related to diverse concentrations of phenol derivatives and mainly CA. The pain reliever profile as well as the neuroprotective properties were characterized. Finally, the pharmacodynamic relevance of nAChRs in rosemary anti-neuropathic activity was assessed.

## Results

### Composition of the rosemary extracts

Aiming to identify the activity of the different components, a fractionation process was applied trying to separate the different group of phenolic compounds typical of rosemary leaves.

**Figure 1:**
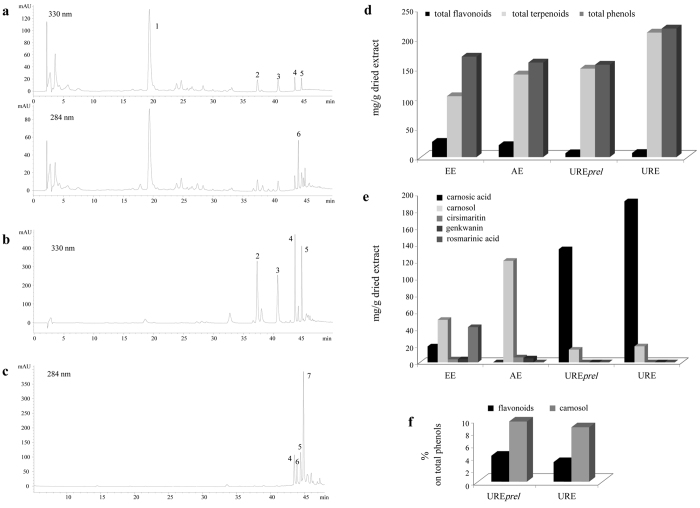
Extracts characterization. HPLC profiles of (**a**) EE at 330 e 284 nm, (**b**) AE at 330 nm and (**c**) URE at 284 nm. 1, rosmarinic acid, 2, cirsimaritin; 3, genkwanin; 4–5, unknown flavonoids; 6, carnosol; 7, carnosic acid. Phenolic distribution in rosemary extracts. (**d**) Comparison among the total contents in flavonoid, terpenoids and total phenols in EE, AE, URE*prel* and URE. (**e**) Quantitative determination of rosmarinic acid and main flavonoid and terpenoid components. (**f**) Percentage of flavonoids and carnosol with respect to total phenols in URE*prel* and URE.

As previously assessed^25^ the total phenolic extract was obtained using ethanol (EE), the acetone extract was applied to selectively recover terpenoids and several lipophilic flavonoids without containing rosmarinic acid (AE); finally, extracts enriched in terpenoids, particularly in CA, were obtained by *n*-hexane extraction assisted by ultrasounds (URE*prel* and URE). The main phenolic structures of rosemary are visible in the Supplementary Figure S1, while representative chromatographic profiles of the extracts are shown in Fig. 1(a–c). EE (Fig. 1a) typically contains all the components that characterize the phenolic fraction: rosmarinic acid and other minor cinnamoyl derivatives, a group of glycosylated and methoxylated flavonoids and the diterpenoid fraction mainly constituted by CA and derivatives^25^. Acetone was useful to prepare an extract characterized by the absence of the polar constituents (mainly rosmarinic acid) and enriched in lipophilic molecules (Fig. 1b). Aimed to obtain a CA-enriched extract, and taking into account that CA undergoes toward rapid oxidation processes when in polar media^26^, the *n*-hexane was chosen as alternative solvent and ultrasounds assisted extraction (UAE) was applied. Noteworthy, a traditional liquid/solid extraction carried out by *n*-hexane was ineffective to recover the terpenoid fraction and specifically CA (data not shown). On the opposite, an unexpected positive result was obtained coupling this solvent with ultrasounds (URE, Fig. 1c).

A comparison of the total phenolic content with the distribution of the different chemical subclasses and some principal constituents is shown in the left panels of Fig. 1(d–f). Although EE, AE and URE*prel* showed a similar concentration of total phenols (Fig. 1d), flavonoids and, particularly, terpenoids levels were consistently different. Progressively, in EE, AE and URE*prel* flavonoids decreased whereas terpenoids increased (Fig. 1d). Regarding URE*prel* and URE, even if prepared applying the same extractive process, the latter sample shared a lower content of flavonoids and carnosol (the main oxidation product of CA), but higher levels of CA (191.3 mg g^−1^ dried extract; Fig. 1e). As depicted in Fig. 1f, URE contained lower levels of flavonoids and carnosol (the main oxidation product of CA) in comparison to URE*prel* as evaluated as percentage on total phenols. Other differences among extracts are shown in Fig. 1e considering single flavonoids (cirsimaritin and genkwanin) and terpenoid (CA and carnosol) constituents. URE and URE*prel* were compared *in vivo* using dosages equivalent as CA concentration. In particular, 100 mg URE*prel* contained 13.5 mg CA, 1.5 mg carnosol, 0.7 mg total flavonoids; 70 mg URE contained 13.5 mg CA, 1.3 mg carnosol, 0.5 mg total flavonoids.

### Effect of repeated administration of EE and AE on CCI-induced hypersensitivity

**Figure 2:**
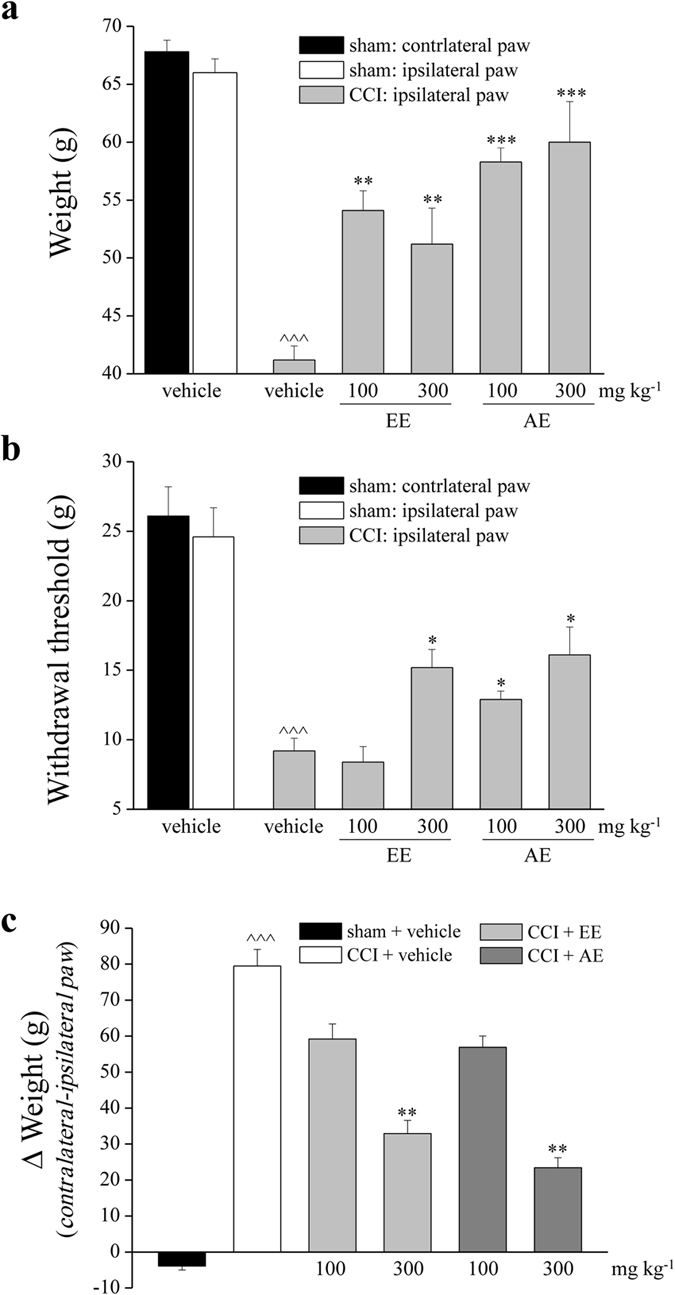
Effects of EE and AE on pain behaviours induced by CCI. (**a**) Sensitivity to a noxious mechanical stimulus as measured by the Paw-pressure test. (**b**) Pain threshold to a non-noxious mechanical stimulus as measured by the Von Frey test. (**c**) Pain assessed by hind limb weight bearing alterations using an Incapacitance test; data are expressed as the difference between the weight applied on the limb contralateral to the injury and the weight applied on the ipsilateral limb. Behavioural tests were performed 14 days after operation, 24h after the last treatment. Vegetal extracts were administered p.o.; control animals were subjected to sham surgery and treated with vehicle. Treatments were performed daily starting on the day of surgery. Each value represents the mean ± SEM of 10 rats per group, performed in 2 different experimental sets. ^^^^^*P* < 0.001 *versus* sham + vehicle; *P < 0.05, **P < 0.01 and ***P < 0.001 *versus* CCI + vehicle.

Fourteen days after sciatic nerve ligation, the weight (noxious mechanical stimulus; Paw pressure test) tolerated on the ipsilateral paw decreased from the value of 66.3 ± 1.1 (sham + vehicle) to 41.2 ± 1.5 g (CCI + vehicle) (Fig. 2a). The response of the contralateral paw was unaltered. Daily administration of EE (100–300 mg kg^−1^) was able to reduce the hypersensitivity (to about 55 g) without a dose response effect. AE (100 and 300 mg kg^−1^) increased pain threshold up to about 60 g. Von Frey test let to evaluate the sensitivity to a mechanical non noxious stimulus, the baseline withdrawal threshold was significantly decreased 14 days after injury on ipsilateral paw of CCI + vehicle group (9.2 ± 0.9 g) in comparison to the control group (24.6 ± 2.1 g) (Fig. 2b). The higher dosage (300 mg kg^−1^) of EE and AE similarly increased the withdrawal threshold of ipsilateral paws, reaching a value of about 16 g, on the contrary only AE maintained the efficacy at 100 mg kg^−1^. As shown in Fig. 2c, the difference between the weight burdened on the contralateral and the ipsilateral paw (Δ weight; Incapacitance test) was increased in CCI + vehicle (79.5 ± 4.6 g) in comparison to sham + vehicle (−3.9 ± 1.1 g) groups. Both treatments with EE and AE (100 mg kg^−1^) reduced by 25% the hind limb weight bearing alterations, the higher dose (300 mg kg^−1^) reduced the Δ weight by 62% (EE) and 70% (AE).

### Effect of repeated administration of URE*prel*, URE and CA on CCI-induced hypersensitivity

**Figure 3:**
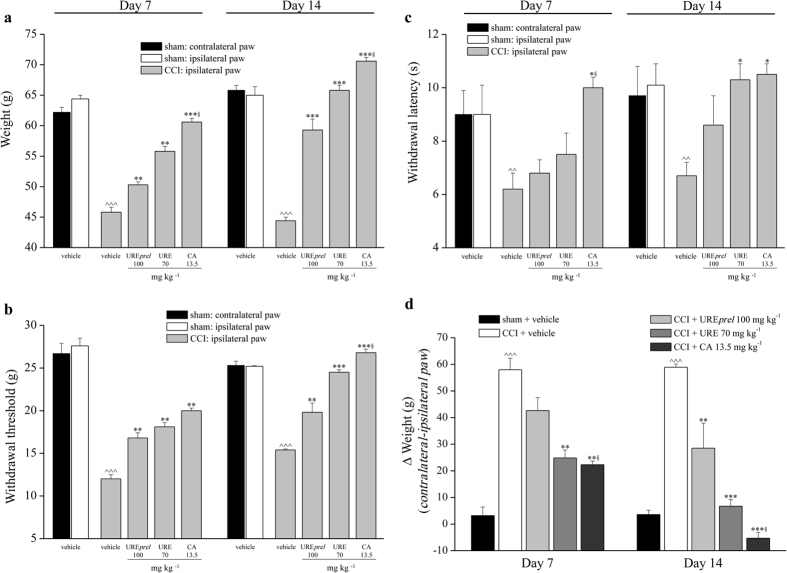
Effects of URE*prel,* URE and CA on pain behaviours induced by CCI. (**a**) Sensitivity to a noxious mechanical stimulus as measured by the Paw-pressure test. (**b**) Pain threshold to a non-noxious mechanical stimulus as measured by the Von Frey test. (**c**) Sensitivity to a noxious thermal stimulus as measured by the Plantar test. (**d**) Pain assessed by hind limb weight bearing alterations using an Incapacitance test. All behavioural tests were performed 7 and 14 days after operation, 24 h after the last treatment. Vegetal extracts and CA were administered p.o. and i.p., respectively; control animals were subjected to sham surgery and treated with vehicle. Treatments were performed daily starting on the day of surgery. Each value represents the mean ± SEM of 10 rats *per* group, performed in 2 different experimental sets. ^^^^^*P* < 0.001 *versus* sham + vehicle; *P < 0.05, **P < 0.01 and ***P < 0.001 *versus* CCI + vehicle; ^§^P < 0.05 versus CCI + URE*prel.*

According to data in Fig. 3, CA was chosen as phytochemical marker to define the dosages of dried samples in order to supply an equivalent CA amount (13.5 mg kg^−1^). On day 7 (after nerve injury), animals tolerated a weight of 45.8 ± 0.8 g in comparison to the of sham + vehicle group (64.4 ± 0.6 g) (Fig. 3a, ipsilateral paw). Repeated administration of URE*prel* (100 mg kg^−1^), URE (70.0 mg kg^−1^) and CA (13.5 mg kg^−1^) significantly reduced mechanical hypersensitivity (Paw pressure test), reaching values of 50.3 ± 0.5 g, 55.8 ± 0.8 g and 60.6 ± 0.6 g, respectively. On day 14, efficacy increased maintaining the order CA > URE > URE*prel*. In particular, URE and CA were able to fully prevent hypersensitivity, CA further increased the threshold up to value higher than control (Fig. 3a). Similar results were obtained by the Von Frey test (Fig. 3b). On Day 7, the withdrawal threshold to mechanical non noxious stimuli of CCI + vehicle rats was decreased to 12.7 ± 0.6 g (ipsilateral paw). Repeated administration of 100 mg kg^−1^ URE*prel* or 70.0 mg kg^−1^ URE increased the pain threshold up to 16.4 ± 0.5 g and 18.1 ± 0.5 g, respectively. CA (13.5 mg kg^−1^) enhanced the effect to 20.0 ± 0.3 g (Fig. 3b). As shown in Fig. 3c, the hypersensitivity to a thermal noxious stimulus (Plantar test) decreased the baseline level of CCI + vehicle animals (9.0 ± 1.1 s; ipsilateral paw) to 6.2 ± 0.6 s on day 7 and 6.7 ± 0.5 s on day 14. CA was the only treatment able to prevent thermal hyperalgesia on day 7 whereas on day 14, URE and CA showed a comparable effect (Fig. 3c). URE*prel* did not induce a significant effect. None of rosemary extracts influenced the pain threshold of the contralateral paw in comparison to the control group (data not shown).

Finally, CCI induced similar hind limb weight bearing alterations induced on days 7 and 14 (Fig. 3d). URE and CA relieved postural imbalance by 57% and 62%, respectively, on day 7 and fully prevented the alteration on day 14. URE*prel* was efficacious on day 14 (Fig. 3d). Significantly different effects induced by CA in comparison to URE *prel* are indicated in Fig. 3; the responses to URE and CA were not significantly different.

**Figure 4:**
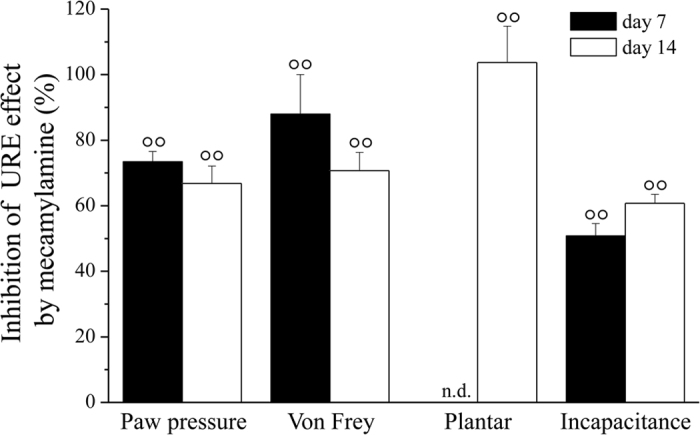
Effects of mecamylamine on the anti-hypersensitive effects URE. Percentage of the inhibition induced by mecamylamine on the effects evoked by URE (considered as 100%) in the Paw-pressure, Von Frey, Plantar and Incapacitance tests. All behavioural tests were performed 7 and 14 days after operation, 24 h after the last treatment. URE was administered daily p.o.; mecamylamine was injected i.p. *bid*. Treatments started on the day of surgery. Each value represents the mean ± SEM of 10 rats *per* group, performed in 2 different experimental sets. °°*P* < 0.01 *versus* CCI + URE without mecamylamine. Absolute values are reported in the Supplementary Figure S2a–d.

Figure 4 shows the percentage of reduction of URE effect in the presence of mecamylamine in the Paw pressure, Von Frey, Plantar and Incapacitance tests. Both on days 7 and 14, mecamylamine prevented URE efficacy by about 60–80%. Absolute values are reported in the Supplementary Figure S2a–d. Mecamylamine did not alter the baseline threshold of the contralateral paw (data not shown).

### Effect of acute administration of URE and CA on CCI-induced hypersensitivity

**Figure 5:**
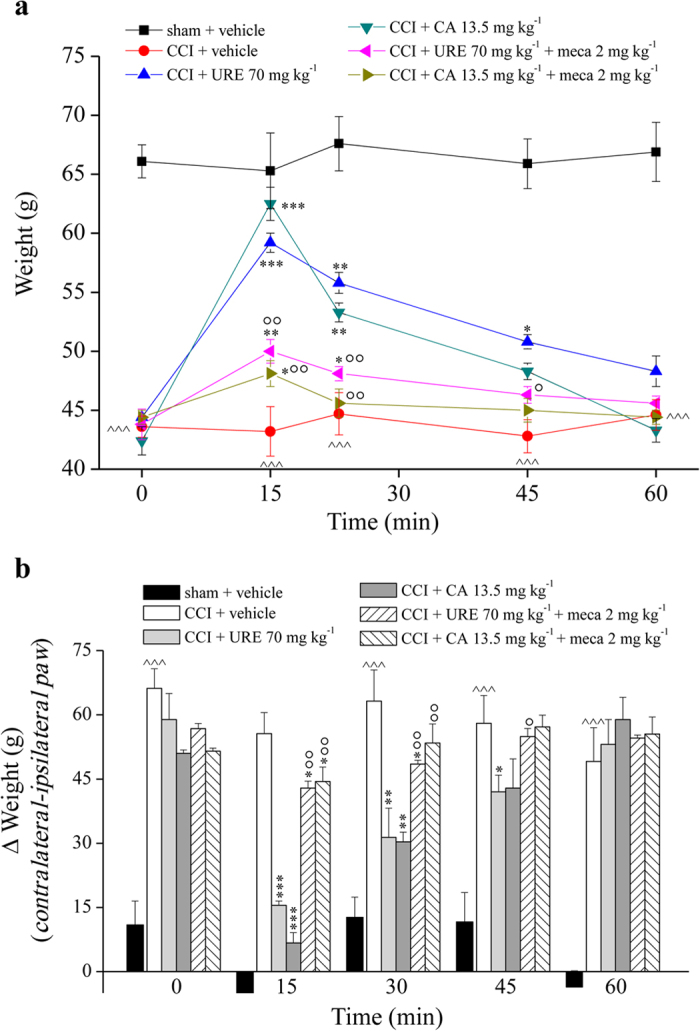
Acute effects of URE and CA on pain behaviours induced by CCI. On day 14 after operation, URE extract (p.o.) and CA (i.p.) were administered in the presence or in the absence of a pre-treatment (−30 min) with mecamylamine (meca; i.p.). (**a**) Sensitivity to a noxious mechanical stimulus as measured by the Paw-pressure test. (**b**) Pain assessed by hind limb weight bearing alterations using the Incapacitance test. Control animals were subjected to sham surgery and treated with vehicle. Each value represents the mean ± SEM of 10 rats *per* group, performed in 2 different experimental sets. ^^^^^*P* < 0.001 *versus* sham + vehicle; *P < 0.05, **P < 0.01 and ***P < 0.001 *versus* CCI + vehicle; °*P* < 0.05 and °°*P* < 0.01 *versus* CCI + URE or CCI + CA.

Effects of a single administration of URE and CA were evaluated over time on day 14 after CCI (Fig. 5a). In the Paw pressure test, 70 mg kg^−1^ URE p.o. reduced mechanical hypersensitivity by 70% peaking 15 min after treatment, at the same time 13.5 mg kg^−1^ CA reduced by 85%. Effects lasted 45 and 30 min for URE and CA, respectively (Fig. 5a). A similar efficacy profile was shown in decreasing the hind limb weight bearing alterations (Incapacitance test; Fig. 5b).

The pre-treatment with mecamylamine (2 mg kg^−1^ i.p., 30 min before URE) significantly reduced the acute anti-hyperalgesic effect of URE (70.0 mg kg^−1^ p.o.) as well as the improvement of postural unbalance. Mecamylamine significantly reverted CA (13.5 mg kg^−1^ p.o.) acute efficacy in both tests (Fig. 5a,b).

### Effect of repeated administration of URE on CCI-induced sciatic nerve damage: histological evaluation

**Figure 6:**
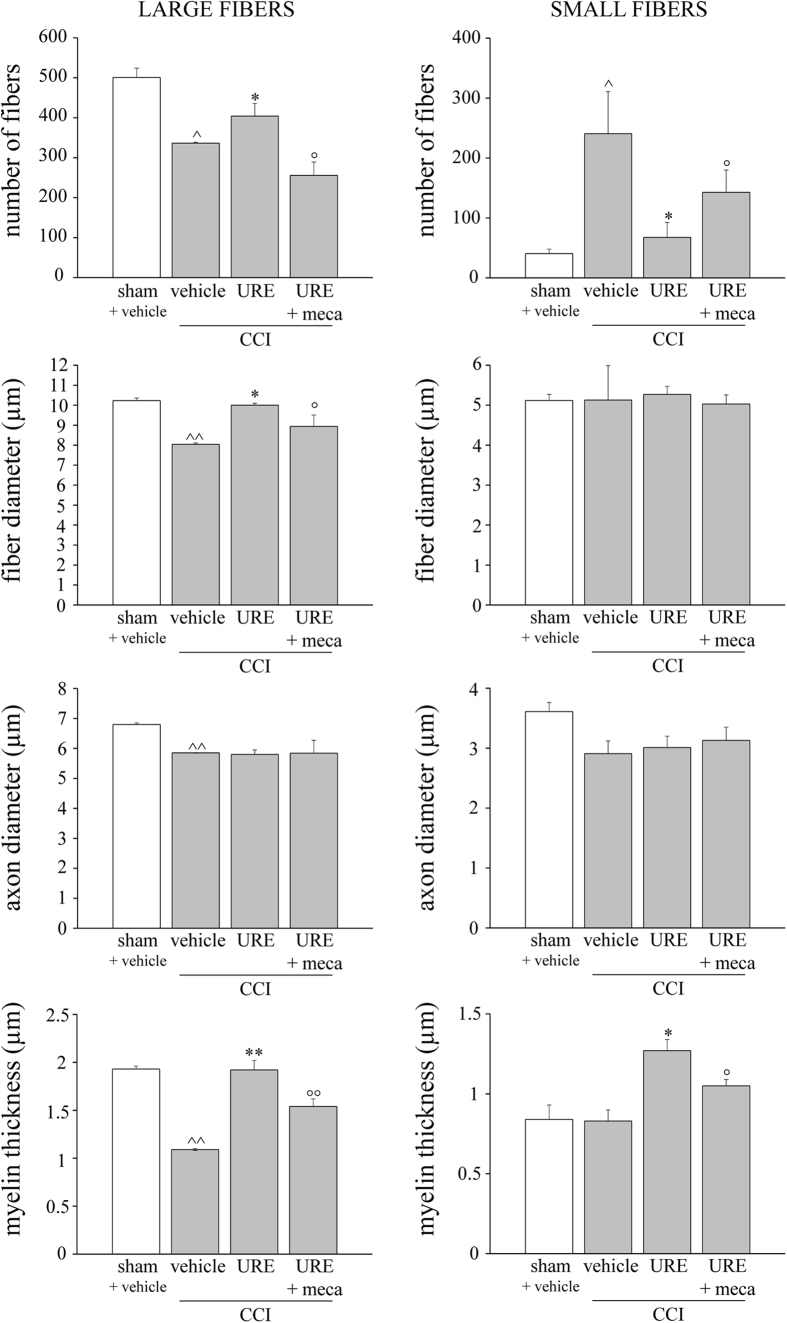
Effects of URE on nerve morphological alterations induced by CCI. URE (70 mg kg^−1^, p.o., daily) and mecamylamine (meca, 2.0 mg kg^−1^, i.p., *bid*) were administered starting on the day of surgery. On day 14, 24 h after the last treatment, 5 μm nerve sections of osmium fixed sciatic nerves collected 900 μm proximally to the injury were analyzed. Ligated nerves (CCI) were compared with nerve of sham-operated animals. Effect of repeated administration of URE + vehicle or URE + mecamylamine on the number of fibers, fiber diameter, axon diameter and myelin thickness of large and small fibers are shown. Quantitative analysis was performed evaluating 6 animals for each group. Each value represents the mean ± SEM of 6 rats *per* group, performed in 2 different experimental sets. ^^^*P* < 0.05 and ^^^^*P* < 0.01 *versus* sham + vehicle; *P < 0.05 and **P < 0.01 *versus* CCI + vehicle; °°*P* < 0.01 *versus* CCI + URE.

Morphometric evaluations on osmium-fixed nerves allowed to measure the number of fibers, fiber diameter, axon diameter and myelin thickness, discriminating between large and small fibers (stratified by diameter in ≥ 6 μm and <6 μm). To better characterize the CCI-induced alterations, 3 distances (300, 900 and 1800 μm) were evaluated starting from the ligation in the proximal part of the nerve (Supplementary Figure S3). On day 14, CCI decreased all nerve structural parameters of large fibers with a gradually less intensity from 300 μm to 1800 μm (Table 1). The number of small fibers increased independently by the distance to ligation (Table 1). Since the significant but middle damage, 900 μm was chosen as optimal nerve distance for evaluating the effect of URE repeated treatment. As depicted in Fig. 6, URE (70 mg kg^−1^ p.o. daily starting from the day of surgery) was able to completely restore the myelin sheet of large fibers. The fiber diameter as well as the number of fibers were also significantly increased. As regards the small fibers, URE increased myelin thickness and reduced the number of fibers. The co-treatment with mecamylamine (2 mg kg^−1^ i.p. *bid* starting from the day of surgery) significantly reduced the protective effect of URE on both large and small fibers (Fig. 6).

**Figure 7:**
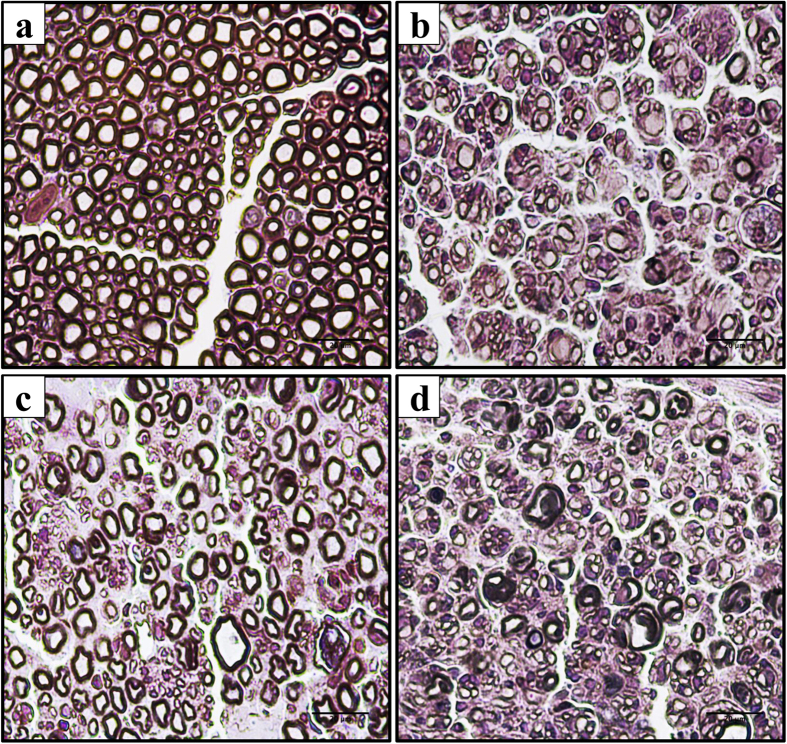
Light micrographs of transverse sections of osmium fixed sciatic nerve after hematoxylin staining. Panels illustrate the effects of URE repeated treatments. (**a**) Sham + vehicle; (**b**) CCI + vehicle; (**c**) CCI + URE; (**d**) CCI + URE + mecamylamine (meca) on day 14. Five μm nerve sections collected 900 μm proximally to the injury were analyzed.

**Figure 8:**
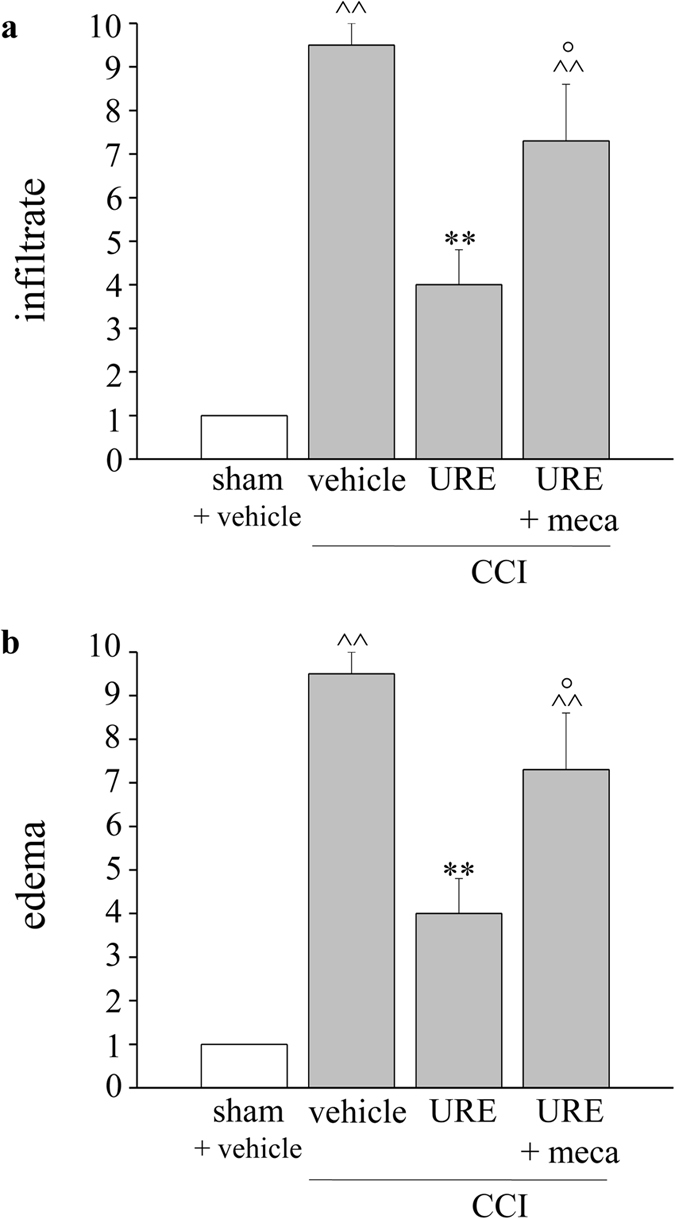
Effects of URE on edema and infiltration of the sciatic nerve induced over 14 days of CCI. Five μm nerve sections of osmium fixed nerves (900 μm proximally to the injury) were hematoxylin stained. (**a**) The presence of inflammatory infiltrate was evaluated and quantified by an arbitrary scale starting from 1, mild infiltrate up to 10, severe infiltrate. (**b**) The presence of edema was evaluated and quantified by an arbitrary scale starting from 1, mild edema up to 10, widespread edema. Effect of repeated treatment with URE (70 mg kg^−1^, p.o., daily) or URE plus mecamylamine (meca, 2.0 mg kg^−1^, i.p., *bid*) was scored in the proximal tract of the ipsilateral ligated nerve and compared with sciatic nerves of sham-operated animals. Semi-quantitative analysis was performed evaluating 6 animals for each group. Each value represents the mean ± SEM of 6 rats *per* group, performed in 2 different experimental sets. ^^^^*P* < 0.01 *versus* sham + vehicle; **P < 0.01 *versus* CCI + vehicle; °*P* < 0.05 *versus* CCI + URE.

As shown in Fig. 7, hematoxylin staining revealed an abundant inflammatory infiltrate and a massive presence of edema among the fibers of CCI animals (900 μm). URE reduced both phenomena by about 50% in a partially mecamylamine-dependent manner (Figs 7 and 8).

**Figure 9:**
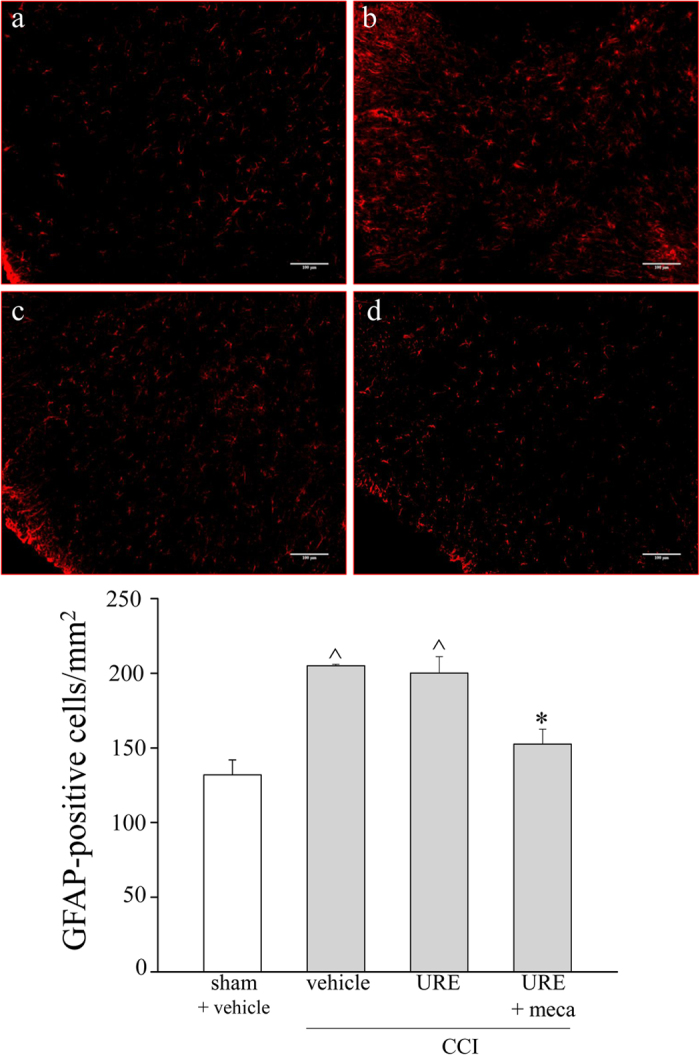
Glial profile in spinal cord scored with GFAP-positive cells 14 days after surgery. URE (70 mg kg^−1^, p.o., daily) and mecamylamine (2.0 mg kg^−1^, i.p., *bid*) were administered starting on the day of surgery. The number of GFAP-positive cells was evaluated in the ipsilateral dorsal horn hemisection of the lumbar tract. In the upper panel, representative transverse sections of spinal cord imaged with 20X objective of (**a**) sham + vehicle; (**b**) CCI + vehicle; (**c**) CCI + URE; (**d**) CCI + URE + mecamylamine (meca). Quantitative analysis of cellular density was performed evaluating 6 animals for each group. Each value represents the mean ± SEM of 6 rats *per* group, performed in 2 different experimental sets. ^^^*P* < 0.05 *versus* sham + vehicle; *P < 0.05 *versus* CCI + vehicle.

The spinal cord was analyzed to assess astroglial cells reorganization 14 days after ligation. Astrocytes were recognized by immunohistochemistry against glial fibrillary acidic protein (GFAP) (Fig. 9a). As shown in Fig. 9b, GFAP-positive cells significantly increased in the superficial laminae of the ipsilateral dorsal horn in CCI rats. The chronic treatment with URE did not modify astrocytes activation. On the contrary, the co-treatment with URE and mecamylamine prevented the increase of GFAP-positive cells in comparison to CCI + vehicle and CCI + URE groups (Fig. 9a,b).

## Discussion

In this report, we showed that extracts from *Rosmarinus officinalis* are able to control pain by inhibiting its progression during a persistent noxious condition. As a pivotal characteristic, rosemary extract prevents damage to the nervous system. Thus, rosemary exerts effects on the origins of neuropathic pain and offers a means to directly modulate nervous signaling. The anti-neuropathic effects are mainly due to the terpenoid fraction in a mecamylamine-reversed manner suggesting a pharmacodynamic role of nAChRs.

In recent years increasing attention is deserved to the therapeutic use of plants also in chronic pathological conditions related to severe alterations of the nervous system^27^. The scientific and clinical relevance of this approach is often compromised by evidences of activity obtained with not standardized procedures and referred to vegetal products scarcely characterized as extraction methods, chemical composition and pharmacodynamic profile. In the present work we obtain a pharmacologically relevant product starting from the historic and popular use of rosemary to relieve pain^28^.

Selected extracts from rosemary leaves have been produced, characterized and compared in terms of phenolic composition to define the more active constituents. EE, AE, URE*prel* and URE contain a progressively decreasing rosmarinic acid and flavonoids component concentration while the terpenoids content increases. In rats underwent the loose ligation of the sciatic nerve, AE shows higher pain relieving efficacy in comparison to EE suggesting the pharmacodynamic role of terpenoids and the minor relevance of rosmarinic acid. Prompted to obtain fractions more enriched in terpenoids, URE*prel* and URE were produced through UAE by *n*-hexane. The extraction method is clean, the low bulk temperature and the rapid execution avoid degradative processes and greatly accelerate the extraction procedure. Generally, the use of ultrasounds offers several advantages in terms of higher yields and higher selectivity, improves the processing time, reduces chemical and physical hazards^29^. Ultrasounds have been recognized for potential industrial application in the phyto-pharmaceutical extraction for a wide range of herbal extracts. To the best of our knowledge, this is the first application of *n*-hexane by UAE to selectively recover the terpenoids from rosemary leaves.

URE*prel* and URE profiles were analyzed *in vivo* employing dosages equivalent in CA content (as a consequence with lower content of flavonoids and carnosol for URE). Repeated administrations were able to reduce the development of CCI-dependent painful response to suprathreshold stimulation and to increase pain threshold to non-noxious stimuli. Both extracts also reduced postural unbalance, a feature of neuropathy progression, as measured by hind limb weight bearing alterations. This measure, in particular, may asses the somatosensory component of neuropathy highlighting spontaneous, non-evoked pain^24^. URE*prel* and URE are more potent and efficacy than AE (300 mg kg^−1^ vs 100 and 70 mg kg^−1^, respectively). The fundamental role of CA was confirmed since 13.5 mg kg^−1^ CA (as standard compound) is fully effective. Nevertheless, differences may be highlighted among URE*prel* and URE and pure CA effects. An improvement of the anti-neuropathic efficacy of these products was obtained by increasing CA concentration (CA > URE > URE*prel*) to the detriment of other components suggesting the possibility that rosemary extracts contains compounds able to partially reduce CA activity. On the other hand, activity differences of CA and carnosol remain to be clarified.

URE and CA shows anti-hypersensitive effect also after a single administration accordingly to previous evidence of acute anti-nociceptive, anti-inflammatory and anti-neuropathic efficacy of rosemary extracts (alcoholic or hydroalcoholic)^4^,^12^, essential oil^30^,^31^ and CA^32^. CA presents anti-inflammatory and anti-oxidant properties since it is able to activate the transcription factor NF-E2-related factor 2 (Nrf2) that in turn mediates transcription of antioxidant/cytoprotective genes by binding to the antioxidant-response element (ARE)^13^. Neuroprotective effects of CA are reported both *in vitro* and *in vivo*^33^,^34^ and the relationship existing between redox unbalance and neuropathy^35^,^36^ suggests the utility of a Nrf2-ARE activator in CCI-induced hypersensitivity. Moreover, CA has been recently described as able to inhibit the AChE activity^16^,^37^ and enhance cholinergic activities^17^. This biological property accounts for the rosemary efficacy against memory impairment and further hints an additional anti-neuropathic mechanisms since the role of the cholinergic system in pain modulation^19^,^38^,^39^. We extensively studied the functions of cholinergic system in pain modulation highlighting the relevance of nAChRs in neuropathic pain relief^22^,^23^,^24^,^40^. In the present results, the nAChR antagonist mecamylamine significantly reduced the anti-hypersensitive effects of both URE and CA indicating a novel pharmacodynamic mechanism for rosemary and its terpenoid component.

The pain-relieving effect of URE and CA progressively increased during treatment suggesting a rescue mechanism that protects nervous tissue from the damages that result in chronic pain. CCI induced morphometric alterations of the sciatic nerve, consistent with previous reports^23^,^24^,^41^. Furthermore, in the present results we analyzed the sciatic nerve at different distances from the injury individuating 900 μm as optimal to evaluate damage characteristics and protective effect promoted by treatments. Repeated administrations of URE preserve number and diameter of nerve fibers and significantly prevents the reduction in both myelin sheet thickness and axonal diameter.

CCI-mediated derangement of nerve morphology is accompanied by a profound local inflammatory reaction that includes edema, infiltration of hematogenous immune cells and the induction of various soluble factors such as cytokines and chemokines^23^,^24^,^41^,^42^. Treatment with URE attenuates the degree of peripheral nerve inflammation, reducing edema and infiltrate suggesting synergy between the anti-inflammatory and the neuroprotective effects of this extract. Accordingly to the neuroprotective properties of nAChR-mediated signals^22^,^23^,^24^,^40^,^43^, the URE neuroconservative effects are strongly impaired by mecamylamine pretreatment.

Finally, damaged peripheral nerves evoke pain-mediating pro-inflammatory and neurotoxic signals in the CNS^44^. Electrical and chemical stimuli promote complex cascades in intracellular transduction pathways that participate in alteration of neuronal and glial functions and subsequently lead to central sensitization^45^. Plasticity of glial cells in spinal cord and brain areas have been recognized as powerful modulators of pain^46^,^47^,^48^, in particular astrocytes may be involved in pain maintenance^46^. In the dorsal horn of CCI rats, we detect an increased density of astrocytes (GFAP-positive cells) which URE does not prevent. This data agrees with previous evidence of the nAChR capability to reduce neuropathic pain and induce neuroprotection concomitantly to glial cell activation^40^,^49^. Since neuroconservation is one of the housekeeping functions of glia^50^ the nAChRs modulation seems to influence the maladaptive plasticity of glial cells reducing pain and favouring neurorestoration. Accordingly, astrocyte activation is reduced in the presence of mecamylamine, the nAChR antagonist able to prevent both the pain relieving and the neuroprotective effects of URE.

As regards the safety profile, oleoresins of rosemary, characterized by a high concentration of these terpenoids, are frequently used as natural food preservative. The EFSA panel has accepted a NOAEL (No Observed Adverse Effect Level) value in the range of 20–60 mg kg^−1^ of carnosic acid plus carnosol in the rat^2^. The ADI (Acceptable Food Intake) has not been defined but the EFSA opinion is that the margin of safety is high enough to conclude that dietary exposure is not of safety concern^2^.

In conclusion, the selected rosemary extract URE, obtained by an innovative extraction process, is able to control pain and prevent alterations of the nervous system induced by nerve injury. The present data offer a preclinical evaluation for a prospective industrial scale up and therapeutic application.

## Material and Methods

### Plant material

Tissue samples were collected from a clone of *Rosmarinus officinalis* L. derived from a mother plant growing at Montebenichi (Florence). This clone is growing in an experimental rosemary clone plantation established in 2010 at the Santa Paolina experimental farm of the Trees and Timber Institute, National Research Council in Follonica (GR), Italy. About 1.5 kg of fresh foliar tissue representing the plant leaf population of the rosemary clone was used for the preparation of the extracts. Aliquot samples of fresh leaves were dried at room temperature in the dark for several days (water content about 45%); both fresh and dried leaves were used for the extraction. In particular, two batches of dried leaves, collected in successive months from the same plants, were used to prepare the *n*-hexane extracts by ultrasounds (URE*prel* and URE) as described below.

### Preparation of the ethanol (EE) and acetone (AE) extracts

For the preparation of the EE extract, fresh leaves (close to 80 g) were dipped in liquid nitrogen and immediately finely grounded in a lab mill. As already described in our previous study^25^ the powder was extracted twice with ethanol alternating magnetic stirring and sonication, with a final yield of 10.5% on dried extract.

AE was obtained extracting dried leaves with acetone for 2 h at room temperature (extractive ratio 1 g/6 mL); the sample was filtered after the extraction, then a base (NH_3_) was added (until pH range 8–9) to deprotonate the carnosic acid and makes the carnosic acid salt. The precipitated impurities were removed by centrifugation and the solution was then acidified (formic acid, pH range 2–3) to induce the precipitation of the terpenoidic fraction recovered by centrifugation^51^. The precipitate was the AE extract with a final yield of 1.6%.

### Preparation of the n-hexane extracts (URE*prel* and URE)

The *n*-hexane extracts were obtained using UAE applied on dried leaves (about 150 g). URE*prel* was from the first batch and URE was obtained from the second batch of dried leaves collected after five months. To minimize the variability we purchased the second batch of fresh material from the same mother plant then applying the same drying process. Concerning the two batches was impossible to guarantee the same ratio young/old leaves. Overall, the harvesting time and the phenological state were the main determinants of the differences between URE*prel* and URE as shown in Fig. 1.

The extraction was carried out by means of a probe system (Danacamerini - Turin) equipped with a titanium horn (Ø = 15 mm) with a conical tip (Ø = 25 mm) working at 19.5 kHz (140 W). Wider tip diameters have less amplitude but can accommodate larger extraction volumes ensuring a more homogeneous sonication; the extractive ratio was 1 g dried leaves/10 mL *n*-hexane with 10 minutes as extraction time. The reproducibility of the extraction was evaluated over time, and both URE*prel* and URE extracts showed a final yield of 6.3% on dried leaves^52^.

### Characterization of the extracts

All the obtained extracts were characterized by HPLC/DAD/MS according to Bellumori *et al*.^53^. Each extract was dissolved in a definite volume of suitable solvent and the clear solutions directly analysed by an HP 1100 L liquid chromatograph equipped with a DAD detector coupled to a TOF mass spectrometer with an electrospray (ESI) interface (Agilent Technologies, Palo Alto, CA, USA).

A Fusion, RP18 column (150 mm × 2 mm i.d., 4 μm) with a precolumn of the same phase (Phenomenex, USA), was used. A multi-step linear solvent gradient was applied with mobile phases (A) 0.1% formic acid/water and (B) CH_3_CN. The method started from 0–15 min 15–25% B; 15–25 min 25–35% B; 25–35 min 35–50% B; 35–40 min 50–100% B with a final plateau of 8 min at 100% B; equilibration time 10 min; flow rate 0.2 mL min^−1^ and oven temperature 26 °C; injection volume 5 μL. The mass spectra were acquired applying the same HPLC method and working in negative and positive way with different energy for fragmentation (150–200 V).

### Quantitative determination

The determination of phenolic compounds was performed using 2 external standards, rosmarinic acid at 330 nm and carnosic acid at 284 nm. The first molecule was also used to quantify the flavonoids, and carnosic acid was used to determine all the other diterpenoids. Calibration curve of rosmarinic acid (purity grade 96% by Sigma-Aldrich) was in a linearity range between 0.1 μg and 9.4 μg with an r^2^ 0.9999; the curve of carnosic acid (purity grade 97% by Sigma-Aldrich) was in the linearity range of 0.05–3.4 μg with an r^2^ 0.9998.

The flavonoid content was also estimated by the calibration curve of genkwanine (purity grade ≥ 95% by Extrasynthese); a five point calibration curve was obtained (range 0 to 0.85 μg) with an r^2^ 0.9999. By comparing the calibration curves of genkwanin and rosmarinic acid at 330 nm, it was estimated that the flavonoids evaluated by the curve of rosmarinic acid give higher values of 68% respect to those from the curve of genkwanin. In this work, to simplify the analytical approach, taking into account that the use of genkwanin to determine all flavonoids is an approximation, and in agreement with our previous studies^25^,^52^,^53^, the flavonoid content was expressed only by the calibration curve of rosmarinic acid.

### Animals

Sprague-Dawley rats (Harlan, Varese, Italy) weighing approximately 200–250 g at the beginning of the experimental procedure, were used. Animals were housed in CeSAL (Centro Stabulazione Animali da Laboratorio, University of Florence) and used at least one week after their arrival. Four rats were housed per cage (size 26 × 41 cm); animals were fed a standard laboratory diet and tap water ad libitum, and kept at 23 ± 1 °C with a 12 h light/dark cycle, light at 7 a.m. All animal manipulations were carried out according to the European Community guidelines for animal care (DL 116/92, application of the European Communities Council Directive of 24 November 1986 (86/609/EEC). The ethical policy of the University of Florence complies with the Guide for the Care and Use of Laboratory Animals of the US National Institutes of Health (NIH Publication No. 85–23, revised 1996; University of Florence assurance number: A5278-01). Formal approval to conduct the experiments described was obtained from the Animal Subjects Review Board of the University of Florence. Experiments involving animals have been reported according to ARRIVE guidelines^54^. All efforts were made to minimize animal suffering and to reduce the number of animals used.

### Induction of peripheral mononeuropathy by CCI

Neuropathy was induced according to the procedure described by Bennett and Xie^55^ Briefly, rats were anaesthetized with 2% isoflurane. Under aseptic conditions, the right (ipsilateral) common sciatic nerve was exposed at the level of the middle thigh by blunt dissection. Proximal to the trifurcation, the nerve was carefully freed from the surrounding connective tissue, and four chromic cat gut ligatures (4–0, Ethicon, Norderstedt, Germany) were tied loosely around the nerve with about a 1 mm spacing between ligatures. After hemostasis was confirmed, the incision was closed in layers. The animals were allowed to recover from surgery and then housed one per cage with free access to water and standard laboratory chow. Another group of rats were subjected to sham surgery in which the sciatic nerve was only exposed but not ligated. The CCI model of mononeuropathy elicits a pain syndrome, characterized by increased response to suprathreshold stimulation, that begins about 3 days after nerve injury and reaches a plateau that lasts between days 7 to 30^24^. Starting from this data, behavioural measurements were performed on days 7 and 14.

### Treatments and measurements

EE and AE extracts were suspended in 1% carboxymethylcellulose sodium salt (CMC) and both administered daily *per os* (p.o.) at the doses of 100 mg kg^−1^and 300 mg kg^−1^ for 13 days, starting from the day of the surgery. Behavioural tests were performed on day 14, 24 h after the last compounds administration. Control rats were treated daily with 1% CMC.

The more lipophilic sample URE*prel* and URE and the pure CA standard (Sequoia Research, UK; purity grade >97%), were suspended in peanut oil and orally (p.o.) administered starting from the day of surgery at the dosages (equivalent in CA concentration) of 100 mg kg^−1^, 70 mg kg^−1^ and 13.5 mg kg^−1^, respectively. Mecamylamine (Sigma-Aldrich, Italy; 2 mg kg^−1^, dissolved in saline solution) was administered *bis in die (bid*) intraperitoneally (i.p.) in co-treatment with URE (30 min before and 8 h after URE administration) for repeated treatment experiments. Behavioural tests were performed at days 7 and 14, 24 h after the last compounds administration. Control animals were treated with vehicles that consisted in peanut oil or saline daily p.o.

Acute treatment experiments were performed on day 14. URE (70 mg kg^−1^) and CA (13.5 mg kg^−1^) were administered p.o.; mecamylamine (2 mg kg^−1^) was administered i.p. 30 min before URE. Control animals were treated p.o. with vehicles.

Behavioral tests were performed 7 or 14 days after surgery using different experimental approaches to evaluate the different clinical components of pain. Mechanical and thermal stimuli were applied to mimic the diverse source of pain in human as well as non noxious and noxious stimuli were used to mimic the clinical perceptions termed allodynia and hyperalgesia, respectively.

### Paw pressure test

The nociceptive threshold in the rat was determined with an analgesimeter (Ugo Basile, Varese, Italy), according to the method published by Leighton *et al*.^56^ and previously extensively described^24^.

### Von Frey Test

The animals were placed in 20 × 20 cm plexiglas boxes equipped with a metallic meshy floor, 20 cm above the bench. A habituation of 15 minutes was allowed before the test. An electronic Von Frey hair unit (Ugo Basile, Varese, Italy) was used following a method previously described^24^.

### Incapacitance test

Weight bearing changes were measured using an incapacitance apparatus (Linton Instrumentation, UK) detecting changes in postural equilibrium after a hind limb injury^58^. The methodology was described in Di Cesare Mannelli *et al*.^24^. Data are expressed as the difference between the weight applied on the limb contralateral to the injury and the weight applied on the ipsilateral one (Δ Weight).

### Plantar test

The Hargreaves radiant heat method^59^ was carried out as modified by Tao *et al*.^60^. The rats were placed individually in clear plastic chambers of Ugo Basile plantar test apparatus for 20 minutes prior to the experiment for the purpose of adaptation. Heat stimulation was applied at IR 60 (infrared intensity 60) on the paw with a 30-second cut-off time. The paw withdrawal latency comprised the time from the start of the beam light until the animal withdrew the paw from the heat stimulus (reaction time) was measured.

### Tissue explant

On day 14, animals were sacrificed and the ipsilateral sciatic nerves, 0.5 cm proximal to the ligation, were explanted; the portion containing the ligature was eliminated. Contralateral nerves and nerves from sham-operated animals were also dissected and equivalent portions were collected.

L4/L5 segments of the spinal cord, exposed from the lumbovertebral column via laminectomy and identified by tracing the dorsal roots from their respective dorsal root ganglia, were collected.

### Osmic acid staining

The sciatic nerve was stored in a 4% glutaraldehyde solution. The tissue samples were osmicated in 1% solution of osmium tetroxide for 2 h under constant agitation. Before and after osmication, the tissue was repeatedly rinsed in 0.1 M sodium cacodylate at pH 7.4. After gradual dehydratation in ethanol, the osmicated nerve samples were embedded in paraffin (Diapath, Milan, Italy). Transverse 5 μm sections were cut on a Reichert microtome (Leica,Rijswijk, The Netherlands), mounted with Canada balsam and observed under a light microscopy.

### Morphometric analysis

Morphometry was performed on cross sections of osmium fixed sciatic nerves 300 μm, 900 μm, and 1800 μm from the ligation in the proximal part. In uninjured control nerves, morphometry was carried out at the corresponding proximal or distal level to the injury. Counts and measurements were performed using an image analysing software (ImageJ 1.48). Micrographs to be analysed were taken using Nikon Olympus BX40 and a 400X objective equipped with NIS F3.00 Imaging Software^®^. Micrographs were randomly selected in non-overlapping areas to cover 50–75% of the total cross-sectional area of the nerve. The total fiber diameter (D), the diameter of the axon (d), and the myelin thickness ([D-d]/2) were measured for each nerve. The number of small fibers, defined as fibers <6 μm, and large fibers, ≥6 μm, was calculated. Since the high degree of degeneration showed by the distal part of the nerve we avoid to report the quantitative measurements of this portion.

### Infiltrate and edema evaluation

Osmium post-fixed sections were stained with hematoxylin and the quantity of infiltrate and edema was evaluated. Images were acquired as previously reported in the section “Morphometric analysis”. Micrographs were randomly selected in non-overlapping areas to cover 50–75% of the total cross-sectional area of the nerve. The sections were semi-quantified by an arbitrary scale starting from 1, mild infiltrate and edema up to 10, severe infiltrate and widespread edema. The procedure was carried out by an independent researcher who was masked to the experiment.

### Immunohistochemical assessment of astrocytes in spinal cord

Fourteen days after surgery, L4/L5 segments of the spinal cord, exposed from the lumbovertebral column via laminectomy and identified by tracing the dorsal roots from their respective DRG, were analyzed to assess astroglial cells. Ten-μm cryostat sections of formalin-fixed spinal cord were incubated with primary antibody directed against GFAP (rabbit, 1:1000; Dako, Glostrup, Denmark) for astrocytes staining o.n. at 4 °C. After rinsing in phosphate-buffered saline with Tween-20, sections were incubated in donkey anti-rabbit immunoglobulin G secondary antibody labeled with Alexa Fluor 568 (1:1000, Invitrogen) at room temperature for 1 h. For all immunohistochemical studies, negative control sections (no exposure to the primary antisera) were processed concurrently with the other sections. Quantitative analysis of GFAP-positive cells was performed by collecting at least 3 independent fields through a 20 × 0.5 numerical aperture (NA) objective. GFAP-positive cells were quantified by means of the automatic thresholding and segmentation features of ImageJ. The GFAP signal in immunostained sections was quantified using FIJI software (distributed by ImageJ, National Institutes of Health, Bethesda, MD, USA) by automatic thresholding images with the aid of the “Moments” algorithm.

### Statistical analyses

Behavioral measurements were performed on 10 rats for each treatment carried out in 2 different experimental sets. For behavioral experiments standard ANOVA followed by Fisher’s protected least significant difference procedure were used. Histological, morphometric and immunohistochemical analyses were performed on 6 rats per group, evaluating 6 sections each of sciatic nerve and spinal cord for each animal. One-way repeated measure ANOVA followed by the Mann–Whitney test was used. All behavioral assessments were made by researchers blinded to rat treatment. Slides from control and experimental groups were labeled with numbers so that the person performing the image analysis was blinded as to the experimental group. In addition, all images were captured and analyzed by an investigator other than the one who performed measurements to avoid possible bias. Data were analyzed using the “Origin 9.0” software (OriginLab, Northampton, MA, USA). Differences were considered significant at a P < 0.05.

## Additional Information

**How to cite this article**: Di Cesare Mannelli, L. *et al*. Anti-neuropathic effects of *Rosmarinus officinalis* L. terpenoid fraction: relevance of nicotinic receptors. *Sci. Rep.* **6**, 34832; doi: 10.1038/srep34832 (2016).

## Supplementary Material

Supplementary Information

Supplementary Figure 1

Supplementary Figure 2

Supplementary Figure 3

Supplementary Figure 4

## Figures and Tables

**Table 1:** Morphometric evaluations of nerve alterations at progressive distances from CCI.

*distance to ligation*	Large fibers					Small fibers				
	*treatment*	number of fibers	fiber diameter	axon diameter	myelin thickness	*treatment*	number of fibers	fiber diameter	axon diameter	myelin thickness
300 μm	sham + vehicle	509.8 ± 23.5	10.67 ± 0.08	6.76 ± 0.03	1.95 ± 0.03	sham + vehicle	23.7 ± 4.5	5.10 ± 0.21	3.59 ± 0.23	0.85 ± 0.11
	CCI + vehicle	273.3 ± 45.3^^^	8.05 ± 0.23^^^^	5.93 ± 0.17^^^^	1.06 ± 0.03^^^^	CCI + vehicle	217.0 ± 50.0^^^	4.80 ± 0.08	3.33 ± 0.18	0.73 ± 0.06
900 μm	sham + vehicle	500.8 ± 19.5	10.23 ± 0.1	6.8 ± 0.05	1.93 ± 0.03	sham + vehicle	40.5 ± 7.6	5.1 2 ± 0.15	3.61 ± 0.15	0.82 ± 0.09
	CCI + vehicle	336.5 ± 2.0^^^	8.04 ± 0.07^^^^	5.85 ± 0.01^^^^	1.09 ± 0.01^^^^	CCI + vehicle	240.7 ± 70^^^	5.13 ± 0.86	2.91 ± 0.21	0.83 ± 0.07
1800 μm	sham + vehicle	505.3 ± 20.2	10.40 ± 0.08	6.70 ± 0.08	1.98 ± 0.05	sham + vehicle	33.7 ± 3.5	5.10 ± 0.21	3.57 ± 0.17	0.83 ± 0.10
	CCI + vehicle	353.5 ± 49.5^^^	9.33 ± 0.43^^^^	6.06 ± 0.13^^^^	1.63 ± 0.14^^^^	CCI + vehicle	143.7 ± 23.7^^^^	4.88 ± 0.10	3.09 ± 0.08	0.89 ± 0.01

Morphometric measurements of number of fibers, fiber diameter, axon diameter and myelin thickness on small and large fibers from sciatic nerves of sham + vehicle and CCI + vehicle (day 14 after surgery). Five μm transverse sections of osmium fixed nerves were analyzed. Each value represents the mean ± SEM of 6 rats per group, performed in 2 different experimental sets. ^^^*P* < 0.05 and ^^^^*P* < 0.01 *versus* sham + vehicle.
