# Supplementary material for: Anti-neuropathic effects of Rosmarinus officinalis L. terpenoid fraction: relevance of nicotinic receptors
Source: Sci Rep. 2016 Oct 7;6:34832. doi: 10.1038/srep34832 (PMC5054390; doi:10.1038/srep34832)
Supplement: Supplementary Information [file srep34832-s1.pdf]

Anti-neuropathic effects of *Rosmarinus officinalis* L. terpenoid fraction: relevance of nicotinic receptors

[a*]Lorenzo Di Cesare Mannelli, [a]Laura Micheli, [a]Mario Maresca, [b]Giancarlo Cravotto, [c]Maria Bellumori, [c]Marzia Innocenti, [c]Nadia Mulinacci, [a]Carla Ghelardini

Supplementary Figure Legends

Figure S1. Some main structures of phenols from rosemary leaves.

Figure S2. Effects of mecamylamine on the anti-hypersensitive effects URE. Peripheral neuropathy was induced by CCI of the right sciatic nerve (ipsilateral). URE (70 mg kg^{-1}, p.o.) was administered daily and mecamylamine (meca, 2.0 mg kg^{-1}, i.p.) was injected *bid*, both starting on the day of surgery. a) Sensitivity to a noxious mechanical stimulus as measured by the Paw-pressure test. b) Pain threshold to a non-noxious mechanical stimulus as measured by the Von Frey test. c) Sensitivity to a noxious thermal stimulus as measured by the Plantar test. d) Pain assessed by hind limb weight bearing alterations using an Incapacitance test. All behavioural tests were performed 7 and 14 days after operation, 24 h after the last treatment. Control animals were subjected to sham surgery and treated with vehicle. Each value represents the mean ± SEM of 10 rats *per* group, performed in 2 different experimental sets. ^^P<0.01 and ^^^P<0.001 *versus* sham + vehicle; **P<0.01 and ***P<0.001 *versus* CCI + vehicle. °°P<0.01 *versus* CCI + URE.

Figure S3. Light micrographs of 5 µm transverse sections of osmium fixed sciatic nerve, illustrating the effects of CCI on day 14. Sections of the proximal portion of CCI + vehicle rats were collected at a) 300 µm; b) 900 µm and c) 1800 µm from injury.
