# Supplementary figures and images for: Anti-neuropathic effects of Rosmarinus officinalis L. terpenoid fraction: relevance of nicotinic receptors
Source: Sci Rep. 2016 Oct 7;6:34832. doi: 10.1038/srep34832 (PMC5054390; doi:10.1038/srep34832)

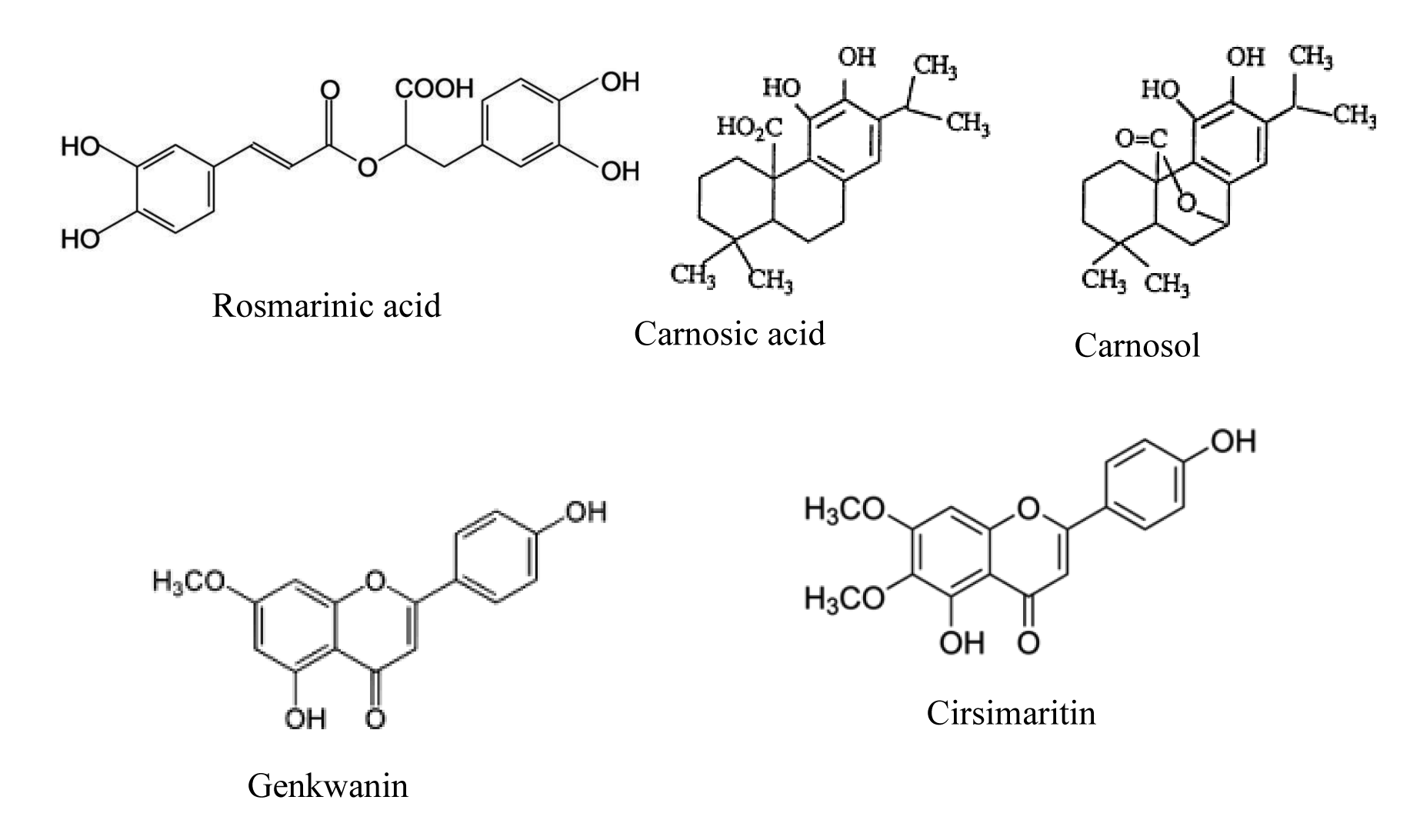

Supplement: Supplementary Figure 1 [file srep34832-s2.tiff]

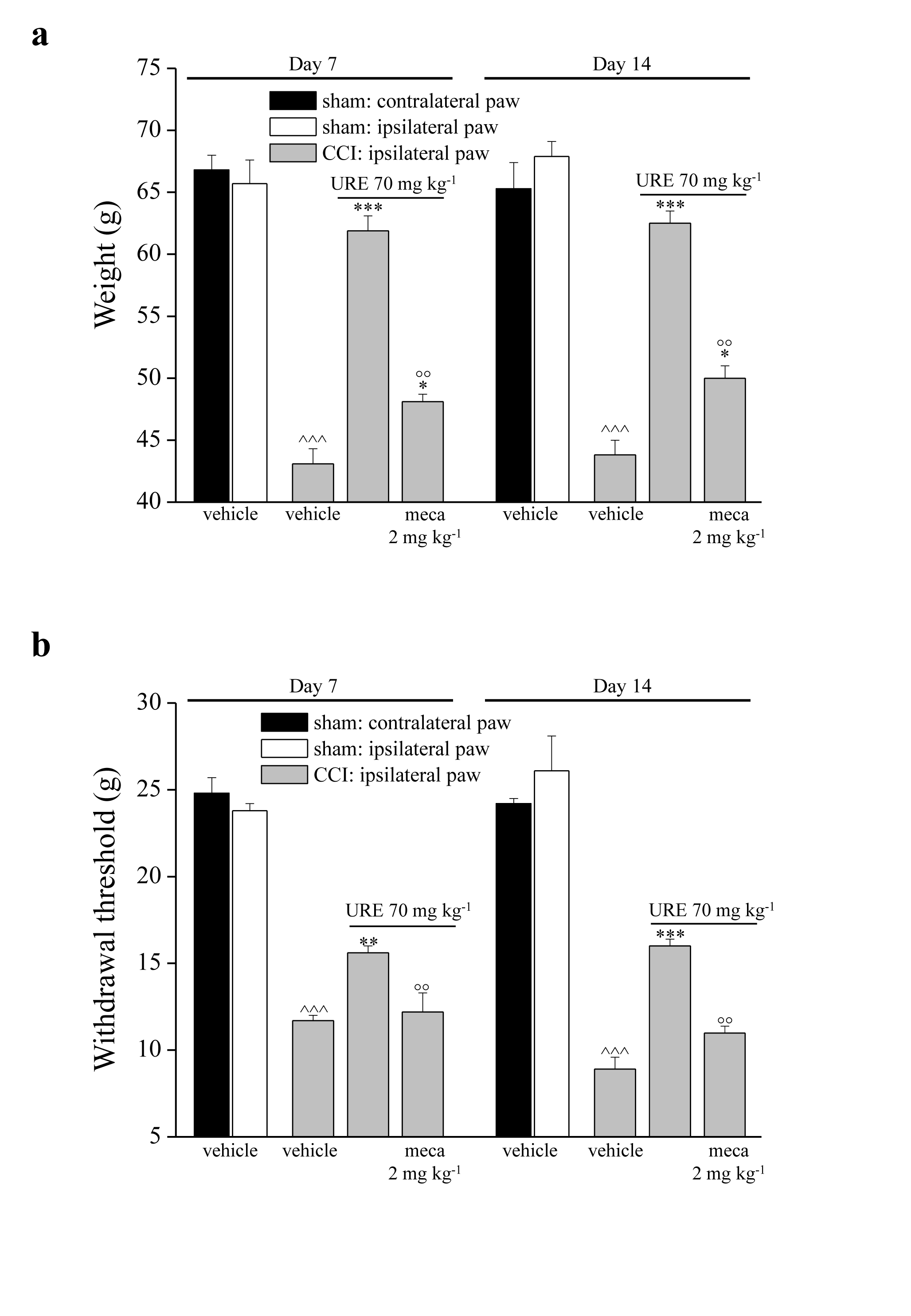

Supplement: Supplementary Figure 2 [file srep34832-s3.tiff]

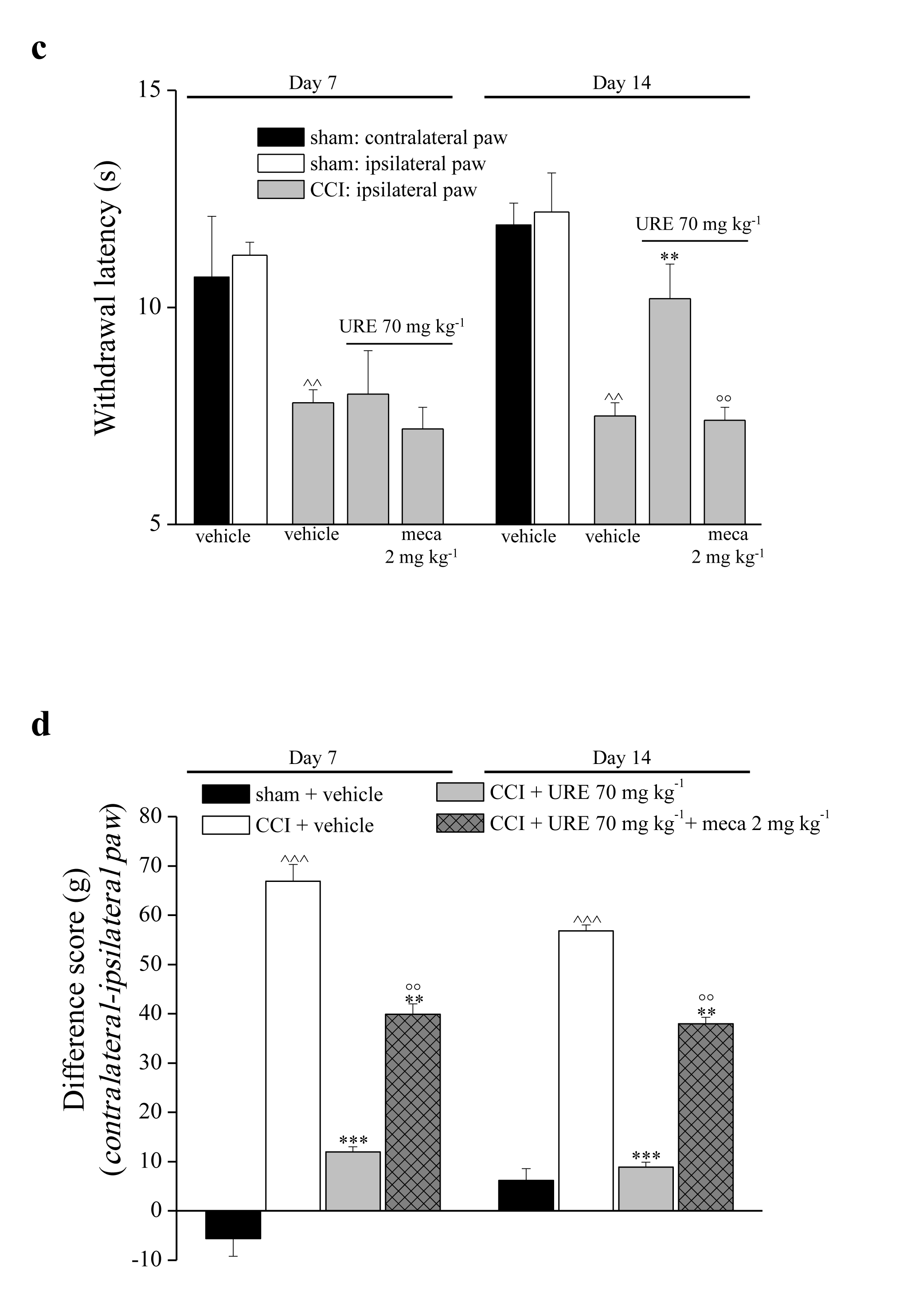

Supplement: Supplementary Figure 3 [file srep34832-s4.tiff]

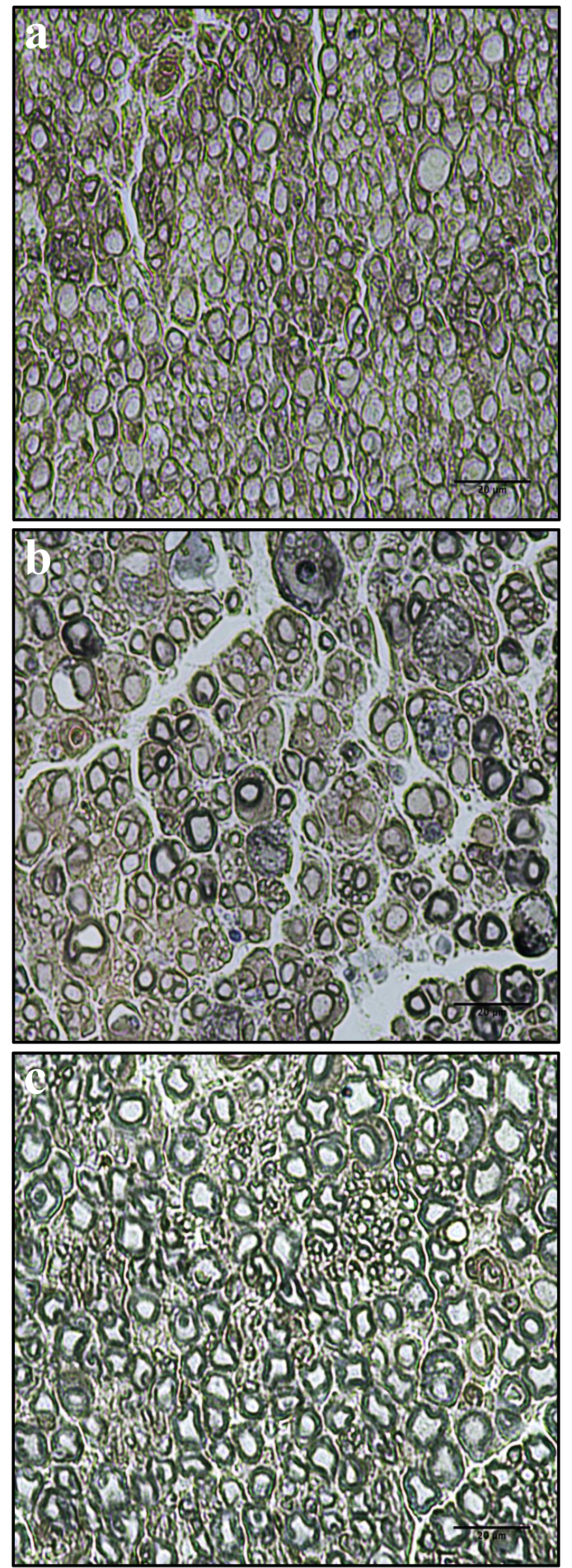

Supplement: Supplementary Figure 4 [file srep34832-s5.tiff]
